# Age-related waning of *in vitro *Interferon-γ levels against r32kDaBCG in BCG vaccinated children

**DOI:** 10.1186/1476-8518-5-8

**Published:** 2007-06-07

**Authors:** B Anuradha, CM Santosh, V Hari Sai Priya, G Suman Latha, KJR Murthy, Valluri Vijaya Lakshmi

**Affiliations:** 1 LEPRA Society – Blue Peter Research Center, Hyderabad, AP, India; 2 Center for DNA Finger printing and Diagnosis, Hyderabad, AP, India; 3 Bhagwan Mahavir Medical Research Centre, Hyderabad, AP, India

## Abstract

**Background:**

*Mycobacterium bovis *BCG vaccine has displayed inconsistent efficacy in different trials conducted in various geographical regions. Nevertheless, it significantly reduces the risk of severe childhood tuberculosis and continues to be used to prevent tuberculosis in many countries. Many studies revealed that efficacy of vaccine wanes with age. Most of the studies were based on *in vivo *and *in vitro *responses to tuberculin. With the advent of newer tests such as *in vitro *interferon-γ assays and identification of potent immunogenic mycobacterial proteins there is a need to corroborate the observations. This study aims at ascertaining the need for a booster at a later age as indicated by *in vitro *release of IFN-γ while evaluating Ag85A as an antigen.

**Methods:**

Ninety healthy children who were without any clinical evidence of the disease, 45 with a BCG-scar and the remaining 45 without scar and 25 with tuberculosis were included in the study. The incidence of TB was analyzed in 216 children attending a DOTS clinic during 1996–2005. CD3+, CD4+ and CD8+ cell counts were measured by Flow cytometry. r32kDaBCG (Ag85A-BCG) protein was used to stimulate T cells in *in vitro *T cell responses and interferon-γ (IFN-γ) cytokine levels in the supernatants were measured by ELISA.

**Results:**

High incidence of TB was observed in age group 13–14 years followed by children in the age group 10–12 years (Chi-square 242.22; p < 0.000). T cell subsets were within the normal range in all subjects. 79% of vaccinated children showed positive proliferative responses with a mean SI value of 4.98 ± 1.99 while only 39% of the unvaccinated and 58% of the tuberculosis children showed positive responses with mean values of 2.9 ± 1.6 (p < 0.001) and 2.9 ± 1.7(p < 0.057), respectively. The stimulation indices in vaccinated children decreased in the older children concurring with an increase in the incidence of TB.

**Conclusion:**

Significantly high levels of *in vitro *IFN-γ demonstrated in BCG vaccinated children in our study substantiate the observation that BCG is effective in children, but the effect may wane with age. The immunity could be boosted using modified r32kDa (Ag85A) of BCG.

## Background

*Mycobacterium bovis *BCG (Bacillus Calmette Guerine) vaccine has displayed inconsistent efficacy in different trials conducted in various geographical regions. Nevertheless, it significantly reduces the risk of tuberculosis by 50% [1] and the risk of severe childhood tuberculosis by 70% [2,3] and continues to be used to prevent tuberculosis in many countries. Moreover, many studies confirm the protective capacity of neonatal BCG against childhood tuberculosis [4-6]. Therefore BCG vaccination at birth must remain a public health priority especially in countries like India with a high incidence of the disease. However, the results of our earlier study [7,8], based on *in vitro *T cell assays using tuberculin as a stimulant, revealed that the effect of BCG wanes with age. Waning of the effect of BCG was also reported by other investigators [9,10], but the results were based mostly on tuberculin skin tests. Although the test has proven to be useful in clinical practice, it has several major limitations. Skin test with tuberculin was not an ideal indicator of BCG vaccination status [7,11-13]. With the advent of newer tests such as in vitro interferon-γ (IFN-γ) assays and identification of potent immunogenic mycobacterial proteins there is a need to corroborate the earlier observations.

Though a variety of live vaccines have been developed as vaccines, until now no booster vaccine has been shown capable of significantly enhancing the level of protective immunity. The efficient recruitment of antigen-specific T-cells principally CD4+ in the lungs, as well the cytokines that are released particularly IFN-γ against *M. tuberculosis *is the sign of improved protective immunity for the development of a new vaccine [14]. IFN-γ is the most important cytokine for inducing the macrophage killing activation mechanism [15].

Preliminary studies conducted at our center demonstrated that 30–34 cluster of culture filtrate protein of *M. bovis *BCG as the most immunogenic [16]. A triad of related gene products 30/32-kDa, referred to as Ag85 complex are the major secretory proteins of *M. tuberculosis *and have been shown to be protective in the guinea pig model of pulmonary tuberculosis. The amino acid sequences of Ag85A and Ag85C are 100% identical for *M. tuberculosis *and *M. bovis *BCG [17]. Their abundant production either extracellulary in broth culture or intracellulary in human monocytes, suggests a vital role in the physiology of the mycobacteria [1]. Studies report that most effective protection was observed when mice were immunized with Ag85A from M. bovis BCG [18]. A single immunization with Ag85 could induce antigen-specific IFN-γ synthesis and more effective protection than live BCG vaccine [19-22].

Reports on 32KDa protein indicating its use as a booster vaccine, were conducted in animals, leading to clinical trials in adult humans. However, there are no reports in children. In India children are vaccinated at birth. This study aims at ascertaining the need for a booster at a later age as indicated by *in vitro *release of IFN-γ while evaluating r32kDa as an antigen.

## Methods

### Subjects

The study, cleared by the Institutional Ethical Committee, included 115 children <12 years of age after obtaining a written consent from the parents. Ninety children who were healthy without any clinical evidence of the disease were included, of these 45 had a scar at the site of BCG administration, and 45 did not have a scar (henceforth referred to as scar-positive and scar-negative groups, respectively). On interrogating the parents, it was recorded that about 10 children (22%) in the latter group had a definite history of vaccination. In addition 25 children with tuberculosis – confirmed by radiological evidence and sputum smear microscopy for pulmonary tuberculosis (n = 11) and biopsy for extra-pulmonary tuberculosis (lymph node; n= 14), visiting the clinics of 'Mahavir Hospital and Research Center' and 'State Tuberculosis Center, AP' were included in the study. Retrospective analysis of data was done to determine the incidence of tuberculosis in 267 children who attended Mahavir Hospital-DOTS clinic, Hyderabad, India during 1996–2005. The population coverage by the clinic was 100000 initially in 1996 and was scaled up to 500000 by 1998. Informed consents and ethical issues were the major constraints in including a larger number of children and performing the tuberculin skin test, which is an invasive procedure.

### Antigen preparation

**Figure 1:**
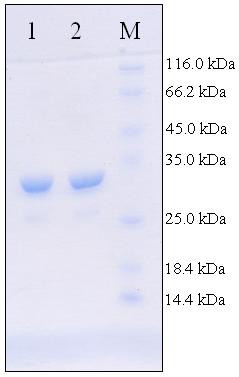
**SDS PAGE representing the purified r32KDa protein**. Lane 1 & 2 represents purified r32KDa protein and M represents molecular weight marker.

Genomic DNA of *M. bovis *was purified from the cultured cells of *M. bovis *BCG using standard procedures. Primers VLV85AFOR: 5'- AATCCGCATATGCAGCTTGTTG ACAGGGTTCGTGGC -3' and VLV85AREV: 5'- AACTGTGGATCCCTAGTGGTGGTGGTGGTGGTGGGCTCCCTGGGGCGCGG -3', designed for the ORF sequence for the *Mycobacterium bovis *gene for 32kDa protein (antigen 85 A) (GenBank: D26486) incorporating a C- terminal His tag. The gene was amplified in GeneAmp PCR System 2700 (ABI) by Acutaq (Sigma) using Genomic DNA as template at different MgCl_2 _(2 mM–8 mM) concentrations. The conditions used for the amplification were: Initial denaturation at 95°C for 10 minutes followed by 40 cycles of 95°C for 1 minute, 65°C for 1 minute and 72°C for 1 minute 30 seconds. This was followed by final extension of 15 minutes at 72°C. The PCR product was extracted and digested with NdeI and BamHI (NEB). This was cloned into pET23a (Novagen). The clones were confirmed by restriction digestion and sequencing with T7 promoter primer on an Applied Biosystems Prism 377 DNA sequencer. The clone was expressed in *E. coli *BL21 (DE3) plysS (Novagen). The transformants were cultured in Terrific broth supplemented with ampicillin (Sigma, Aldrich, St.Louis, MO, USA) & chloramphenicol (Sigma, Aldrich, St.Louis, MO, USA) and was induced at late log phase with 0.7 mM IPTG for 6 hours. The protein was purified under native conditions using Ni-NTA(Qiagen, Valencia, CA, USA) Chromatography, designed for the purification recombinant 6XHis-tagged proteins and was concentrated using 3 KDa Molecular Weight cut off Centriplus concentrators (Millipore, Billerica, MA, USA). Integrity of the protein was checked on 10% SDS PAGE (Fig. 1). The protein concentration was estimated by Bradford's method.

### CD3+, CD4+ and CD8+ cell counts

CD3+, CD4+ and CD8+ cell counts were performed using four-color BD FACSCalibur system flow cytometer (BD Biosciences, San Jose, CA, USA). For staining, 50 μl of whole blood was aliquoted into 12 × 75 mm polystyrene BD TruCOUNT™ Tubes (BD MultiTEST™, San Jose, CA, USA) containing 20 μl CD3 FITC/CD8 PE/CD45 Per CP/CD4 APC antibody (BD Pharmingen™, Franklin Lakes, NJ, USA). The samples were mixed well and incubated for 20 minutes at room temperature in the dark and then lysed using 450μl of lysing solution and incubated for 10 minutes. The tubes were centrifuged at 90 g (1000 rpm) for 5 minutes and the supernatant discarded. Two ml of PBS buffer (PH 7.2–7.4, 0.05 M) was added in the tube and it was centrifuged again at 90 g (1000 rpm) for 5 minutes. After the supernatant was discarded and the cells resuspended in 500ul of PBS, the samples were acquired within 2 hrs after staining and anlysed by using CellQuest Pro software (BD Biosciences, San Jose, CA, USA).

### PBMC assay

For assessing T cell proliferation, blood was drawn in Heparin (5000 I.U/5 ml; Biological E limited, Hyderabad, AP, India), diluted with equal volume of RPMI-1640 medium (Invitrogen corporation, Grand Island, N.Y. USA) without AB serum, layered on Histopaque (Sigma, St Louis, MO, USA) gradient in 1:3 proportion and centrifuged at 200–350 g (1500–2000 rpm) for 30 minutes. After the peripheral blood mononuclear cells (PBMC) were isolated, washed twice to remove the cell debris and platelets each at 90 g (1000 rpm) for 10 minutes, the cell concentration was adjusted to 1 million cells/ml. Viability of the cells was checked using Trypan blue (Sigma Aldrich, St.Louis, MO, USA). To 0.1 ml of cell suspension 0.1 ml of media for the control wells, 8 μl (3 mg/ml) of recombinant protein (r32kda-BCG, also referred to as Ag85A-BCG) in 0.1 ml of media and 30 μl of 1 mg/ml Concanavalin A (Con-A, Sigma Aldrich, St.Louis, MO, USA) for the experimental wells were added in duplicates. Concentration of the recombinant protein was standardized based on the optimal proliferation of the cells using children's blood samples. The plate was incubated at 37°C with 5% CO_2 _and humidified air. At the end of the 3^rd ^day for Con-A and 5^th ^day for the antigen, MTT (3-(4-5-dimethyl thiazol-2-yl) 2,5, diphenyl tetrazoleum bromide) (Sigma Aldrich, St.Louis, MO, USA) was added and optical density (OD) measured at 570 nm and 620 nm reference filter [3,23] in ELISA reader (BIO-RAD, Hercules, CA, USA). Stimulation index (SI), a ratio between the OD values of the test and control, was considered as positive if ≥ 2 [23].

### *In vitro *interferon gamma assay

Sandwich Enzyme Linked Immunosorbent Assay (ELISA) was performed to measure the IFN-γ levels in the supernatants collected from the above cultures. To each well, 50 μl of the capture antibody (BD pharmingen™, Franklin Lakes, NJ, USA). diluted in coating buffer was added and incubated over night at 4°C. After 5 washes, the wells were blocked with 50 μl of 2% BSA for 3 hours at 37°C. A further 5 washes were followed by the addition of 50 μl of supernatants and standards and then incubated for 4 hours at room temperature. After another 5 washes, 50 μl secondary antibody was added, incubated for 3 hours at 37°C and washed 5 times. After addition of 50 μl of working detector (HRP antigen, BD pharmingen™, Franklin Lakes, NJ, USA) and incubation for one and half hour and 5 washes, the wells were incubated with the substrate (50 μl) Ortho Phenyl Diamine (OPD, Sigma, St. Louis, MO, USA) + H_2_O_2 _(Qualigen, Mumbai, MH, India) for 20 minutes at room temperature in the dark. 50 μl of stop solution was added and absorbance was measured at 490 nm.

### Statistical analysis

Student's-t test was used for comparing the mean values between the groups. Chi-square test to compare number of children between the groups was done by cross tabulation method. A p value of ≤ 0.05 was considered as significant.

## Results

### Expression and purification of M. bovis BCG r32KDa protein

The protein was purified under native conditions using Ni-NTA Chromatography. The purified r32KDa protein was separated on an 10% gel and visualized for expected protein band using Coomassie Brilliant Blue. The purity of the protein was about 95%. To remove the endotoxin contamination, purified recombinant 32KDa protein was incubated with 10% v/v polymyxin B-agarose (Sigma-Aldrich; binding capacity, 200 to 500 μg of LPS from Escherichia coli serotype O128:B12/ml) for 1 hour at 4°C. For further immunological studies this endotoxin free r32KDa protein was used.

### Cell counts and proliferation

The mean (±SD) numbers of CD3+, CD4+, CD8+ cells and the SIs in T cell proliferation assay against Con-A, were comparable in all the three groups of children (values not shown in the tables).

### Age groups

When the 267 children with TB (tuberculosis)were classified into different age-groups, the highest number (n = 116) was observed in age group 13–14 years followed by children in the age group 9–12 years (n = 96). The numbers declined further with the last group (age < 6 years) having the least number (n = 20). Chi-Square between the number of children in different age groups was highly significant (242.22; p < 0.000).

**Figure 2:**
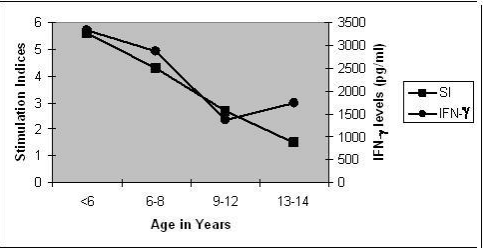
**T-cell assays with BCG r32kDa: Stimulation Indices and Interferon-γ levels in different age-groups of BCG-vaccinated children (n = 45)**. Definition of abbreviation: IFN-γ = Interferon – gamma, SI = Stimulation Index. **Statistical Significance between mean values of IFN-γ levels (pg/ml): **(a) < 6 years & 9–12 years (3316 ± 718 & 1360 ± 344; p < 0.003). (b) 6–8 years and 9–12 years (2880 ± 733 & 1360 ± 344; p < 0.01). **Statistical Significance between mean values of SI: **(a)<6 years & 9–12 years (5.69 ± 2.21 & 2.70 ± 1.13; p < 0.0002) (b) 6–8 years & 9–12 years (4.36 ± 2.10 & 2.70 ± 1.13; p < 0.013)

Stimulation Indices and their IFN-γ levels in the supernatants in PBMC assay according to their age distribution in vaccinated children were shown in Figure 2. Significant differences were observed between the SI in <6 years (5.60 ± 2.8) and that in 9–12 years groups (2.70 ± 1.13) (p < 0.0002); between 6–8 years (4.36 ± 2.1) and 9–12 years (2.70 ± 1.13) groups (p < 0.013). Significant differences were observed between the IFN-γ levels in <6 years (3316 ± 718 pg/ml) and that in 9–12 years groups (1360 ± 344 pg/ml) (p < 0.003); between 6–8 years and 9–12 years groups (2880 ± 733 and 1360 ± 344; p < 0.01).

### PBMC assay

Percent number and mean ± SD of negative (<2) and positive (≥2) stimulation indices (SI) in PBMC assays with BCGr32Kda protein observed in children (a) with BCG scar (b) without scar and (c) with TB are shown in Table 1. The proliferation in healthy BCG scar positive children were significantly high when compared to that in scar negative- and tuberculosis- children with a p < 0.0004 and 0.0014 respectively. Seventy nine percent of vaccinated children showed positive proliferative responses with a mean SI value of 4.98 ± 1.99 while only 39% of the unvaccinated and 58% of the tuberculosis children showed positive responses with mean values of 2.9 ± 1.6 and 2.9+1.7, respectively. Chi-square test by cross tabulation method to compare number of children between scar positive and scar negative children was: 10.465; p < 0.001 and between scar positive versus tuberculosis, 3.630; p < 0.057.

**Table 1:** Percent-number and mean ± SD of Stimulation-Indices in PBMC assays with BCGr32Kda protein in different categories of children.

	**Scar +ve** **n = 45**	**Scar -ve** **n = 45**	**TB** **n = 25**	**P value**
**Mean (± SD) of positive SIs**	4.9 ± 1.9^a^	2.9 ± 1.17^b^	2.9 ± 0.73^c^	p < 0.0004^ab^ p < 0.0014^ac^
**Mean (± SD) of negative SIs**	1.6 ± 0.29	1.6 ± 0.31	1.7 ± 0.20	
%n with positive SI*	79	39	58	
%n with negative SI*	21	61	42	

Stimulation Indices (SI) < 2: negative & ≥ 2: positive in T cell assays.

Statistical analysis of the mean values of SIs was done by Student's t-test.

Chi-square test to compare number of children was done by cross tabulation method:

*Scar positive Vs. Scar negative, χ^2 ^= 10.465; p < 0.001

* Scar positive Vs. Tuberculosis, χ^2 ^= 3.630; p < 0.057

### *In vitro *IFN-γ assay

**Figure 3:**
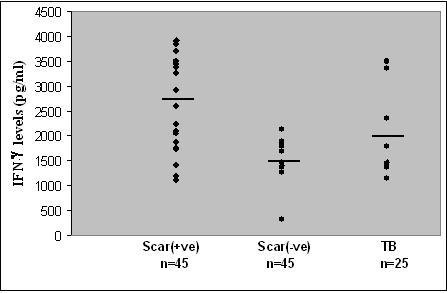
**IFN-γ levels in the supernatants of T cell assays stimulated with BCG r32KDa protein in children**. Definition of abbreviation: IFN-γ = Interferon – γ. Horizontal bars represent mean values. **Statistical Significance between mean values of IFN-γ levels (pg/ml): **Scar positive Vs. Scar negative: p < 0.0008

The overall mean IFN-γ levels (2744 ± 1004pg/ml) in vaccinated children was significantly high (p < 0.0008) when compared to 1556 ± 490 pg/ml in scar-negative and to 2112 ± 988 pg/ml in TB children (Figure 3). 2046 pg/ml of IFN-γ was considered as the cut-off value (based on the least arithmetic mean + standard deviation in scar negative children). 66% of the vaccinated children, 40% of TB children and only 10% of scar negative children showed IFN-γ levels greater than 2046 pg/ml.

## Discussion

An understanding of protective immune responses in humans is essential for the rational development and clinical testing of new, effective vaccines [24] which are characterized by bacterial antigen specific lymphoproliferative responses, IFN-γ production, high Th1 type-Th2 type ratios and induction of CTL [25]. It is important to understand the quantitative relationship between host responses and disease and to identify proliferation of T cells specific to antigen, which contribute to protective immunity. *In vitro *cytokine production by peripheral blood mononuclear cells can be an important and reliable measure of immunocompetence. Also, spontaneous release of cytokines by PBMCs may serve as a measure of their activation *invivo *[26]. In the present study proliferative responses and *in vitro *IFN-γ production were thus demonstrated in different categories of children from Hyderabad, India. The exposure to environmental mycobacteria is presumed to be more or less similar in all the children since they are from same geographical location. Comparison has been made between scar positive and scar negative children to evaluate the influence of BCG vaccination on the assay.

It was observed in this study that BCG induced immunity wanes with age, concurring with an increase in the number of children with tuberculosis. Waning of immunity is of particular public health interest because it may result in increased susceptibility later in life. The mechanism underlying the gradual loss of effectiveness of BCG as the individual reaches 10 to 15 years of age is poorly understood [27,28]. In the study on tuberculosis epidemiology in south India (1961–68), the prevalence of infection over the study period varied between 1 to 2.1%, 6.4 to 7.9% and 15.4 to 16.9% in the 0–4, 5–9 and 10–14 year age group, respectively [29]. Similar observations were made in other parts of India [30,31]. Studies reported that memory immunity slowly declines but can be recovered by boosting if a candidate antigen that can be specifically recognized by this immunity is reintroduced [27]. Therefore, pre-exposure priming with a highly efficacious attenuated vaccine strain should be followed by post exposure boosting with a potent subunit vaccine [9]. The results observed in earlier and present studies probably necessitate the need for a booster vaccine perhaps at the age of about four years, much before the waning begins.

The low responses observed in children without a BCG-scar in this study, further reiterate that Ag85A may be effectual not only as a booster-vaccine but also as an antigen in *in vitro *assays as a correlate of protection. A possible explanation for children with tuberculosis exhibiting moderate *in vitro *immune responses as observed in this study may be the presence of *M. tuberculosis *specific T cells. Thus, Ag85A protein may also be a potential diagnostic agent for assessing the immune status of healthy individuals and predict their clinical outcome.

Furthermore, the nature of the cells responding to *M. tuberculosis *infection and how their relative contribution changes over time is a crucial aspect in vaccine development [32]. Animal models suggest that CD4+ T cells are the most important aspect of the protective response in primary infection [33,34]. Though the subset of T cells that released IFN-γ was not identified in this study, there are reports that BCG stimulates both CD4^+ ^and CD8^+ ^cells *in vitro*, while PPD stimulates only CD4^+ ^lymphocytes [35]. CD8^+ ^T cells which also respond by releasing IFN-γ[36] exhibit strong recognition of Ag85A from healthy controls [37]. Intranasal vaccination with *Mycobacterium tuberculosis *antigen85A against airway *Mycobacterium tuberculosis *challenge in mice induced a significantly higher level of interferon-γ [38]. Further studies should involve the specific memory cells and their kinetics.

Though it has been demonstrated earlier that Ag85A of *Mycobacterium tuberculosis *is immunogenic, the studies were conducted in animals or adults. A recombinant modified vaccinia virus Ankara expressing this antigen (MVA85A) has already entered into clinical trials in 2002 [39]. To conclude, the present study involved antigen 85A of BCG and was based on observations in children vaccinated with BCG at birth. Given their age, it is unlikely that the children had a prior exposure to environmental mycobacteria, significantly high *in vitro *IFN-γ levels observed in BCG vaccinated children in our study substantiate that BCG is effective in children, but that the effect may wane with age. The immunity could be boosted using modified r32kDa (Ag85A) of BCG.

## Authors' contributions

AB carried out the study, collection of samples, performed all the experiments, statistical analysis of data and prepared the manuscript. SCM synthesized r32kDa protein. HSP was associated in standardizing techniques. SLG supervised sample collection and oversaw clinical documentation. KJRM and VVL were involved in the conception of the study. KJRM contributed towards the clinical aspects. VVL designed the study and edited the manuscript. All authors read and approved the final manuscript.
